# Effect of chewing gum combined with WeChat-enhanced instruction on bowel preparation in constipated patients: a randomized–controlled trial

**DOI:** 10.1093/gastro/goaf034

**Published:** 2025-04-28

**Authors:** Cong Gao, Deli Zou, Weiyi Wang, Yingchao Li, Jie Han, Dongshuai Su, Xingshun Qi

**Affiliations:** Department of Gastroenterology, General Hospital of Northern Theater Command, Shenyang, Liaoning, P. R. China; Department of Gastroenterology, General Hospital of Northern Theater Command, Shenyang, Liaoning, P. R. China; Department of Gastroenterology, General Hospital of Northern Theater Command, Shenyang, Liaoning, P. R. China; Department of Medical and Nursing, Dalian Rehabilitation Recuperation Center, Dalian, Liaoning, P. R. China; Department of Nursing, The Second Affiliated Hospital of Army Medical University, Chongqing, P. R. China; Department of Gastroenterology, General Hospital of Northern Theater Command, Shenyang, Liaoning, P. R. China; Department of Gastroenterology, General Hospital of Northern Theater Command, Shenyang, Liaoning, P. R. China; Department of Gastroenterology, General Hospital of Northern Theater Command, Shenyang, Liaoning, P. R. China; Department of Gastroenterology, General Hospital of Northern Theater Command, Shenyang, Liaoning, P. R. China

**Keywords:** chewing gum, WeChat-enhanced instruction, bowel preparation, constipation

## Abstract

**Background and aims:**

Constipated patients have higher risk of poor bowel preparation and suffer from dysfunction of the intestinal motor. Chewing gum can stimulate gut motility and enhanced instructions can improve the quality of bowel preparation. The objective of this study was to investigate whether chewing gum combined with WeChat-enhanced instruction can increase the quality of bowel preparation in constipated patients.

**Methods:**

This was a single-center, endoscopist-blinded, randomized–controlled trial. Patients were assigned (1:1) to the chewing gum and WeChat-enhanced instruction (CGW) group and the control group. Patients in both groups received 3 L of polyethylene glycol (PEG) before colonoscopy. Patients in the CGW group were asked to chew one piece of gum for 20 min after drinking each 1 L of PEG and received enhanced instruction via WeChat the day before colonoscopy. The quality of the bowel preparation (primary outcome), adenoma and/or polyp detection rate (ADR/PDR), number of polyps and/or adenomas, procedure time, and adverse events were compared.

**Results:**

A total of 115 patients were finally analysed, including 60 in the CGW group and 55 in the control group. The proportion of adequate bowel preparation and the Boston Bowel Preparation Scale score were not statistically different between the two groups (76.7% vs 70.9%; 6.80 ± 1.42 vs 6.40 ± 1.78; both *P *>* *0.05). There was no significant difference in the ADR/PDR and number of polyps and/or adenomas (both *P *>* *0.05). However, there was a significantly higher incidence of nausea in the CGW group than in the control group (33.3% vs 16.4%, *P *=* *0.036).

**Conclusions:**

Chewing gum combined with WeChat-enhanced instruction does not improve the quality of bowel preparation for colonoscopy in constipated patients but does increase the incidence of nausea.

## Introduction

Colorectal cancer (CRC) is the third-most common cancer worldwide [1]. The incidence rate of CRC has been increasing in China [2]. Colonoscopy is the most common approach for CRC screening [3]. Up to one-fifth of participants undergoing colonoscopy have constipation [4]. The quality of the bowel preparation has an effect on colonoscopy performance. Notably, patients with constipation have a lower quality of bowel preparation than those without [5]. Thus, it is of great importance to improve the bowel-preparation quality in such patients.

Constipated patients suffer from dysfunction of the intestinal motor and secretory activity [6]. It seems that sham feeding can activate the cephalic–vagal axis and stimulate the colonic motility and secretory activity [7]. Chewing gum is a convenient approach of sham feeding. However, whether chewing gum can improve bowel-preparation quality remains controversial. A previous randomized trial suggested that chewing gum added to high-dose senna before colonoscopy improved the quality of the bowel preparation [8]. In contrast, another randomized–controlled trial (RCT) failed to show any benefit of chewing gum in bowel cleaning, but it can improve patients’ satisfaction with the process of bowel preparation [9]. Additionally, few studies have investigated its effectiveness in constipated patients.

Poor compliance is another reason for inadequate bowel preparation [10]. In our previous study, short-message-service-enhanced instructions have improved the quality of bowel preparation probably via increasing the patients’ compliance [11]. Recently, WeChat has been the most widely used social software in China. Accordingly, it has been considered to be a more convenient approach for enhanced instructions instead of a short-message service.

In the current study, we aimed to evaluate whether chewing gum combined with WeChat-enhanced instruction can improve the quality of bowel cleansing as an adjunct to bowel preparation in constipated patients undergoing colonoscopy.

## Methods

### Study design and participants

A single-center, endoscopist-blinded RCT was conducted between February 2022 and August 2023 at the Department of Gastroenterology of the General Hospital of Northern Theater Command (Shenyang, P. R. China). The study protocol conformed to the ethical guidelines of the 1975 Declaration of Helsinki. This trial was approved by the Medical Ethical Committee of the General Hospital of Northern Theater Command (approval number Y [2023] 177) and registered at ClinicalTrials.gov (identifier NCT05447403). All patients provided written informed consent at the time of the colonoscopy appointment.

Inclusion criteria were as follows: (i) age ≥18 years; (ii) patients undergoing colonoscopy; (iii) diagnosis of constipation (according to the Rome IV criteria [12]); and (iv) written informed consent.

Exclusion criteria were as follows: (i) history of colorectal surgery; (ii) major psychiatric disorders; (iii) pregnancy or breastfeeding; (iv) contraindications for colonoscopy (e.g. heart failure, renal insufficiency); (v) suspicion of intestinal obstruction, stenosis, or perforation; (vi) allergy to gum ingredients and polyethylene glycol (PEG); or (vii) WeChat not available or could not be used by the patients themselves or their family members.

### Randomization and blinding

Study group assignment was based on a computer-generated table of random numbers that was created by C.G. with random-numbers-generator software (Qi He Inc., Wu Han, P. R. China). Allocation concealment was achieved by using opaque sealed envelopes that contained the random numbers. During the process of the colonoscopy appointment, patients were assigned (1:1) to the chewing gum and WeChat-enhanced instruction (CGW) group and the control group by a nurse who was not involved in the acquisition and analysis of the data.

Patients were asked to refrain from discussing the bowel preparation with endoscopists and assistant nurses. Blinding of the patients was not possible due to the differences between the two bowel-preparation regimens.

### Bowel-preparation regimens

Patients were informed to eat a semi-liquid and low-slag diet (which refers to very little dietary fiber or hard muscle fiber contained in the food) for breakfast and lunch, and a full-liquid diet for dinner on the day before the colonoscopy, and to fast on the day of the colonoscopy. A split-dose regimen of 3 L of PEG was used for all colonoscopies: patients were asked to take 1 bag of PEG with 1 L of water at 21:00 on the day before the colonoscopy, and the remaining two bags of PEG with 2 L of water at 4:00 and 30 mL of simethicone at 6:00 on the day of the colonoscopy as previously described [11, 13]. If a patient underwent colonoscopy in the afternoon, the remaining 2 L of PEG and simethicone were permitted to be taken at noon. If a patient felt that the bowel-preparation quality was inadequate after traditional bowel preparation, an additional bag of PEG could be taken. Even if a patient did not strictly obey the bowel-preparation regimen, we would discuss with him or her about whether the colonoscopy could be performed. Patients in the CGW group were asked to chew one piece of xylitol sugarless gum (Extra, Mars Inc., Guangzhou, P. R. China) for ∼20 min after drinking each 1 L of PEG.

### Instructions

At our department, standard written instructions were provided for all patients, including: (i) colonoscopy appointment time; (ii) dietary guidance; and (iii) time and method for consuming the laxative. Patients in the CGW group received enhanced instruction via WeChat the day before the colonoscopy, including dietary guidance, instructions for laxative intake and chewing gum, and the importance of adequate bowel preparation. Patients in the control group would not receive any further enhanced instruction via WeChat.

### Outcomes

The primary outcome was the quality of the bowel preparation, which was assessed by using the Boston Bowel Preparation Scale (BBPS) [14]. A total BBPS score of ≥6 with a BBPS score of ≥2 for each colon segment was considered to show adequate bowel preparation.

Secondary outcomes were as follows: (i) adenoma and/or polyp detection rate (ADR/PDR): proportion of patients with at least one adenoma and/or polyp detected during colonoscopy; (ii) number of polyps and/or adenomas; (iii) cecal intubation rate; (iv) insertion time: the time required to intubate the colonoscope to the cecum; and (v) withdrawal time.

### Adverse events

Adverse events during or after bowel preparation were assessed before the colonoscopy procedure, including abdominal pain, bloating, nausea, and vomiting.

### Sample-size calculation

A previous study reported that an adequate bowel-cleansing rate in the WeChat-enhanced instruction and the conventional instruction group was 89.8% and 66.4%, respectively [15]. Considering a type I (α) error of 5%, a type II (1-β) error of 10%, and a dropout rate of 20%, it was determined that 75 patients would be required in each group.

### Statistical analysis

Continuous variables were expressed as means ± standard deviations and compared by using the Student’s *t*-test. Categorical variables were summarized as absolute frequencies and percentages, and analysed by using the chi-squared test or Fisher’s exact test for expected frequencies of <5. *P*-values of <0.05 were considered statistically significant. All analyses were performed by using SPSS software (Ver. 27.0; SPSS Inc., Chicago, Illinois, USA).

## Results

### Baseline characteristics

**Figure 1.**
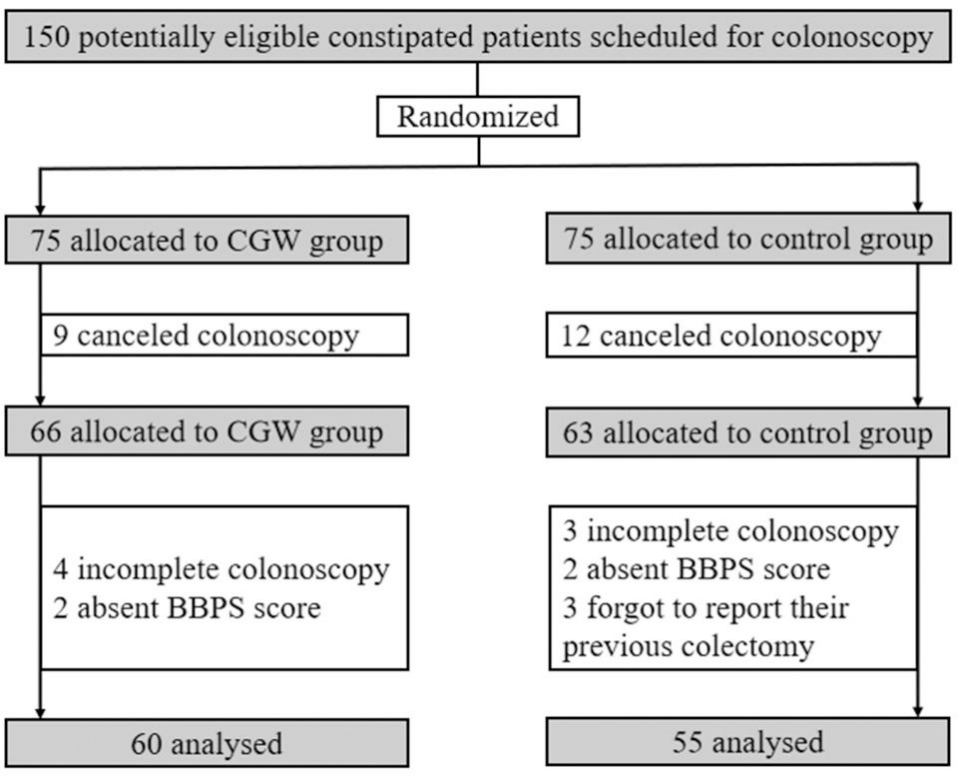
Flowchart of patients’ enrollment. CGW = chewing gum and WeChat-enhanced instruction, BBPS = Boston Bowel Preparation Scale.

Among the 150 patients who were enrolled in this study, 21 canceled the colonoscopy. Besides, three patients forgot to report their previous colectomy, which were found during the colonoscopy, four patients lacked a BBPS score, and cecal intubation failed in seven patients due to colonic obstruction or intolerable pain. Finally, 115 eligible individuals were included in the final analysis. A flow diagram that describes the patient enrollment is shown in Figure 1.

No significant differences were found between the CGW and control groups in terms of baseline characteristics (Table 1). Except for constipation, reasons for colonoscopy were abdominal pain (*n *=* *15) and bloating (*n *=* *5).

**Table 1.** Comparison of baseline characteristics between CGW and control groups

Variables	CGW (*n *=* *60)	Control (*n *=* *55)	*P*-value
Age, years, mean ± SD	53.28 ± 15.39	51.51 ± 16.67	0.554
Male	15 (25.0)	18 (32.7)	0.360
BMI, kg/m², mean ± SD	22.87 ± 3.40	22.63 ± 3.45	0.715
Colonoscopy time			0.140
Morning	42 (70.0)	45 (81.8)	
Afternoon	18 (30.0)	10 (18.2)	
Prior colonoscopy	21 (35.0)	21 (38.2)	0.723
Sedated colonoscopy	7 (11.7)	2 (3.6)	0.166
Prior abdominal surgery	19 (31.7)	18 (32.7)	0.903

CGW = chewing gum and WeChat-enhanced instruction, SD = standard deviation, BMI = body mass index.

### Primary outcome

The percentages of adequate bowel preparation between the CGW and control groups were not significantly different (76.7% vs 70.9%, *P *=* *0.312). There was no significant difference in the percentages of adequate bowel preparation for right, transverse, and left colon segments between the CGW and control groups (76.7% vs 70.9%, *P *=* *0.312; 96.7% vs 89.1%, *P *=* *0.109; 96.7% vs 87.3%, *P *=* *0.062).

No significant differences were found between the CGW and control groups in terms of BBPS scores (6.80 ± 1.42 vs 6.40 ± 1.78, *P *=* *0.184) (Table 2). The differences in the mean scores for right, transverse, and left colonic regions between the CGW and control groups were also insignificant (1.97 ± 0.66 vs 1.75 ± 0.58, *P *=* *0.061; 2.42 ± 0.56 vs 2.27 ± 0.71, *P *=* *0.227; 2.40 ± 0.56 vs 2.35 ± 0.75, *P *=* *0.662).

**Table 2.** Comparison of primary and secondary outcomes between CGW and control groups

Variables	CGW (*n *=* *60)	Control (*n *=* *55)	*P*-value
Primary outcomes			
Adequate bowel preparation, *n* (%)			
Total	46 (76.7)	39 (70.9)	0.312
Right colonic region	46 (76.7)	39 (70.9)	0.312
Transverse colonic region	58 (96.7)	49 (89.1)	0.109
Left colonic region	58 (96.7)	48 (87.3)	0.062
BBPS score, mean ± SD			
Total	6.80 ± 1.42	6.40 ± 1.78	0.184
Right colonic region	1.97 ± 0.66	1.75 ± 0.58	0.061
Transverse colonic region	2.42 ± 0.56	2.27 ± 0.71	0.227
Left colonic region	2.40 ± 0.56	2.35 ± 0.75	0.662
Secondary outcomes			
ADR/PDR, *n* (%)	33 (55.0)	29 (52.7)	0.807
Number of polyps and/or adenomas, mean ± SD	1.3 ± 1.9	1.2 ± 1.6	0.356
Insertion time, min, mean ± SD	8.32 ± 4.61	7.63 ± 4.15	0.421
Withdrawal time, min, mean ± SD	9.50 ± 4.79	8.86 ± 5.56	0.520

CGW = chewing gum and WeChat-enhanced instruction, BBPS = Boston Bowel Preparation Scale, SD = standard deviation, ADR/PDR = adenoma and/or polyp detection rate.

### Secondary outcomes

The ADR/PDR was 55.0% in the CGW group and 52.7% in the control group (*P *=* *0.807). There was no significant difference in the number of polyps and/or adenomas between the CGW and control groups (1.3 ± 1.9 vs 1.2 ± 1.6, *P *=* *0.356). Only a few patients had diverticulosis (3 of 115, 2.6%) and protuberant or inflammatory lesions (7 of 115, 6.1%) (Table 2).

The rates of successful cecal intubation were comparable between the two groups (CGW group vs control group, 93.8% vs 94.8%, *P *=* *0.965). The reasons for incomplete colonoscopy included colorectal space-occupying lesions (CGW group vs control group, 5% vs 0%, *P *=* *0.245) and intolerable discomfort (CGW group vs control group, 3.3% vs 5.5%, *P *=* *0.459). There was no significant difference in the insertion and withdrawal times between the CGW and control groups (8.32 ± 4.61 vs 7.63 ± 4.15 min, *P *=* *0.421; 9.50 ± 4.79 vs 8.86 ± 5.56 min, *P *=* *0.520).

### Adverse events

Regarding adverse events, the proportions of abdominal pain, bloating, or vomiting were comparable between the two groups (*P *=* *1.000, *P *=* *1.000, *P *=* *0.275, respectively). However, the incidence of nausea was higher in the CGW group than in the control group (33.3% vs 16.4%, *P *=* *0.036) (Table 3).

**Table 3.** Comparison of adverse events between CGW and control groups

Variables	CGW (*n *=* *60)	Control (*n *=* *55)	*P*-value
Abdominal pain	2 (3.3)	1 (1.8)	1.000
Bloating	3 (5.0)	3 (5.5)	1.000
Nausea	20 (33.3)	9 (16.4)	0.036
Vomiting	6 (10.0)	2 (3.6)	0.275

CGW = chewing gum and WeChat-enhanced instruction, SD = standard deviation.

## Discussion

The findings of this trial show that chewing gum combined with WeChat-enhanced instruction does not achieve a statistically significant improvement in the quality of bowel preparation before colonoscopy but does increase the incidence of nausea in constipated patients.

Patient factors are important determinants of suboptimal bowel preparation. Especially, one of the most common factors associated with bowel-preparation quality is constipation [16] and constipated patients have a higher risk of inadequate bowel preparation [5]. Several RCTs have evaluated the efficacy of stronger bowel-preparation regimens in constipated patients. The published RCTs usually choose the regimen of laxatives plus prokinetic drugs [17] or additional laxatives [18–20] and the control groups usually choose laxatives such as PEG [17–19] or sodium phosphate [20]. But the results of these RCTs are conflicting. Overall, insufficient evidence exists to provide an appropriate protocol for bowel preparation in these patients. In this setting, we attempted to explore the efficacy of chewing gum combined with WeChat-enhanced instruction for bowel preparation in this randomized–controlled endoscopist-blinded trial. Notably, whether chewing gum or WeChat-enhanced instruction can improve the quality of bowel preparation is still controversial among studies. One trial evaluated the efficacy and safety of high-dose senna plus chewing gum for bowel preparation before colonoscopy and found that chewing gum enhanced bowel cleansing [8]. But it should be noted that the regimen used for bowel cleansing was high-dose senna, which is uncommon in clinical practice. Another previous study showed that chewing gum could not improve the quality of bowel preparation, which was consistent with the findings of our study [9]. But it should be noted that the chewing-gum protocol was not common, in which patients in the gum group were asked to chew one piece of gum every 2 h until their colonoscopy examination, and the mean number of pieces of gum consumed was 2.8 ± 0.8. By comparison, our study employed split-dose bowel preparation and designed a more widely used protocol of chewing gum, in which patients chewed three pieces of gum in total to more accurately demonstrate the effect of chewing gum on bowel cleansing before colonoscopy. Besides, this previous study showed an improvement in patient satisfaction with the process of bowel preparation [9], which was contradictory to our findings that chewing gum increased the risk of nausea, probably due to the fact that patients were in an empty state during bowel preparation. Chewing gum may increase the secretion of gastric acid and gastrointestinal symptoms, such as nausea, especially in patients with gastritis, gastric ulcers, and chronic pharyngitis.

In the present study, the total BBPS score, the BBPS score of each colonic segment, and the percentage of adequate bowel preparation of all and each colonic segment were higher in the CGW group than in the control group, although the difference was not of statistical significance. However, we found that patients in the CGW group had a higher incidence of nausea than patients in the control group, which was in contrast to the results of earlier studies [8, 9, 21]. This indicates that chewing gum as an adjunctive treatment to a split dose of 3 L of PEG may not be suitable for constipated patients, primarily because they have already had dysfunction of intestinal motility. There is currently no effective choice for the radical treatment of constipation and most patients with constipation can only use laxatives to temporarily relieve symptoms in clinical practice. Therefore, a higher stimulus to activate gut motility would aggravate gastrointestinal symptoms, such as nausea.

This is the first RCT to assess the effect of chewing gum combined WeChat-enhanced instruction for bowel preparation in constipated patients. Our study has several limitations. There was a high cancelation rate for colonoscopy (14%), probably due to the prevention and control measures during the COVID-19 epidemic. As we used xylitol gum in the current trial and the effect of a sweet taste could not be completely excluded, this may be a variable that could have affected our current results. Lastly, it is hard for us to completely monitor whether each patient strictly chewed the gum for 20 min or no more than one piece of gum following each 1 L of PEG.

## Conclusions

Chewing gum combined with WeChat-enhanced instruction in this single-center, endoscopist-blinded RCT of constipated patients did not improve the quality of bowel preparation before colonoscopy, but did increase the incidence of nausea. Although chewing gum is simple and inexpensive, whether it could be used in addition to a split-dose PEG preparation before colonoscopy needs further discussion. Modified regimens should be investigated with the aim to improve the quality of bowel preparation before colonoscopy in patients with constipation.

## Authors’ contributions

X.Q. was responsible for conceptualization; C.G. and X.Q. were responsible for data analysis; C.G., Y.L., J.H., D.S. and X.Q. were responsible for data curation; C.G., W.W. and X.Q. were responsible for drafting; C.G., W.W., Y.L., J.H., D.S., D.Z. and X.Q. were responsible for revising the manuscript. D.Z. and X.Q. were responsible for the study supervision. All authors have read and approved the final version of the manuscript.

## References

[1] Sung H , Ferlay J, Siegel RL et al Global cancer statistics 2020: GLOBOCAN estimates of incidence and mortality worldwide for 36 cancers in 185 countries. CA Cancer J Clin 2021;71:209–49. 33538338 10.3322/caac.21660

[2] Li N , Lu B, Luo C et al Incidence, mortality, survival, risk factor and screening of colorectal cancer: a comparison among China, Europe, and northern America. Cancer Lett 2021;522:255–68. 34563640 10.1016/j.canlet.2021.09.034

[3] Davidson KW , Barry MJ, Mangione CM et al; US Preventive Services Task Force. Screening for colorectal cancer: US Preventive Services Task Force recommendation statement. JAMA 2021;325:1965–77. 34003218 10.1001/jama.2021.6238

[4] Gimeno-GARCíA AZ , Baute JL, Hernandez G et al Risk factors for inadequate bowel preparation: a validated predictive score. Endoscopy 2017;49:536–43. 28282690 10.1055/s-0043-101683

[5] Gandhi K , Tofani C, Sokach C et al Patient characteristics associated with quality of colonoscopy preparation: a systematic review and meta-analysis. Clin Gastroenterol Hepatol 2018;16:357–69.e10. 28826680 10.1016/j.cgh.2017.08.016

[6] Camilleri M , Ford AC, Mawe GM et al Chronic constipation. Nat Rev Dis Primers 2017;3:17095. 29239347 10.1038/nrdp.2017.95

[7] Roslan F , Kushairi A, Cappuyns L et al The impact of sham feeding with chewing gum on postoperative ileus following colorectal surgery: a meta-analysis of randomised controlled trials. J Gastrointest Surg 2020;24:2643–53. 32103455 10.1007/s11605-019-04507-3 PMC7595968

[8] ERGüL B , Filik L, KOçAK E et al Efficacy and safety of gum chewing in adjunct to high-dose senna for bowel cleansing before colonoscopy: a single-blind randomized controlled trial. Saudi J Gastroenterol 2014;20:356–9. 25434316 10.4103/1319-3767.145325 PMC4271010

[9] Fang J , Wang S-L, Fu H-Y et al Impact of gum chewing on the quality of bowel preparation for colonoscopy: an endoscopist-blinded, randomized controlled trial. Gastrointest Endosc 2017;86:187–91. 27327849 10.1016/j.gie.2016.05.051

[10] Zhang Y , Wang L, Wu W et al Predictors of inadequate bowel preparation in older patients undergoing colonoscopy: a systematic review and meta-analysis. Int J Nurs Stud 2024;149:104631. 37963423 10.1016/j.ijnurstu.2023.104631

[11] Gao C , Chen H, Cao R et al Impact of enhanced instructions by short message service on the quality of bowel preparation for colonoscopy. Acta Gastroenterol Belg 2022;85:406–7. 35709788 10.51821/85.2.9989

[12] Lacy BE , Mearin F, Chang L et al Bowel Disorders. Gastroenterol 2016;150:1393–407.e5. 10.1053/j.gastro.2016.02.031 27144627

[13] Cao RR , Wang L, Gao C et al Effect of oral simethicone on the quality of colonoscopy: a systematic review and meta-analysis of randomized controlled trials. J Dig Dis 2022;23:134–48. 35075814 10.1111/1751-2980.13084

[14] Lai EJ , Calderwood AH, Doros G et al The Boston bowel preparation scale: a valid and reliable instrument for colonoscopy-oriented research. Gastrointest Endosc 2009;69:620–5. 19136102 10.1016/j.gie.2008.05.057 PMC2763922

[15] Wang S-L , Wang Q, Yao J et al Effect of WeChat and short message service on bowel preparation: an endoscopist-blinded, randomized controlled trial. Eur J Gastroenterol Hepatol 2019;31:170–7. 30418256 10.1097/MEG.0000000000001303

[16] Mahmood S , Farooqui SM, Madhoun MF. Predictors of inadequate bowel preparation for colonoscopy: a systematic review and meta-analysis. Eur J Gastroenterol Hepatol 2018;30:819–26. 29847488 10.1097/MEG.0000000000001175

[17] Tajika M , Niwa Y, Bhatia V et al Efficacy of mosapride citrate with polyethylene glycol solution for colonoscopy preparation. World J Gastroenterol 2012;18:2517–25. 22654449 10.3748/wjg.v18.i20.2517 PMC3360450

[18] Parente F , Vailati C, Bargiggia S et al 2-Litre polyethylene glycol-citrate-simethicone plus bisacodyl versus 4-litre polyethylene glycol as preparation for colonoscopy in chronic constipation. Dig Liver Dis 2015;47:857–63. 26232311 10.1016/j.dld.2015.06.008

[19] Li Y , Jia X, Liu B et al Randomized controlled trial: Standard versus supplemental bowel preparation in patients with Bristol stool form 1 and 2. PLoS One 2017;12:e0171563. 28241037 10.1371/journal.pone.0171563 PMC5328251

[20] Pereyra L , Cimmino D, González MC et al Colonic preparation before colonoscopy in constipated and non-constipated patients: a randomized study. World J Gastroenterol 2013;19:5103–10. 23964144 10.3748/wjg.v19.i31.5103 PMC3746382

[21] Lee J , Lee E, Kim Y et al Effects of gum chewing on abdominal discomfort, nausea, vomiting and intake adherence to polyethylene glycol solution of patients in colonoscopy preparation. J Clin Nurs 2016;25:518–25. 26818376 10.1111/jocn.13086

